# Changes in error-correction behavior according to visuomotor maps in goal-directed projection tasks

**DOI:** 10.1152/jn.00121.2021

**Published:** 2022-03-23

**Authors:** Ayane Kusafuka, Ryoji Onagawa, Arata Kimura, Kazutoshi Kudo

**Affiliations:** ^1^Department of Life Science, Graduate School of Arts and Sciences, The University of Tokyo, Tokyo, Japan; ^2^Department of Sports Research, Japan Institute of Sports Sciences, Tokyo, Japan

**Keywords:** error-correction, motor variability, multisensory integration, visual perturbation, visuomotor map

## Abstract

Humans can move objects to target positions out of their reach with certain accuracy by throwing or hitting them with tools. However, the outcome—the final object position—after the same movement varies due to various internal and external factors. Therefore, to improve outcome accuracy, humans correct their movements in the following trial as necessary by estimating the relationship between movement and visual outcome (visuomotor map). In the present study, we compared participants’ error-correction behaviors to visual errors under three conditions, wherein the relationship between joystick movement direction and cursor projection direction on the monitor covertly differed. This allowed us to examine whether the error-correction behavior changed depending on the visuomotor map. Moreover, to determine whether participants maintain the visuomotor map regardless of the visual error size (cursor projection) and proprioceptive errors (joystick movement), we for the first time focused on whether temporary visual errors deviating from the conventional relationship between joystick movement direction and cursor projection direction (i.e., visual perturbation) are ignored. The visual information was occasionally perturbed in two ways to create a situation wherein the visual error was larger or smaller than the proprioceptive error. We found that participants changed their error-correction behaviors according to the conditions and could ignore visual perturbations. This suggests that humans can be implicitly aware of differences in visuomotor maps and adapt accordingly to visual errors.

**NEW & NOTEWORTHY** We found that participants changed their error-correction behaviors according to the conditions and could ignore visual perturbations. This suggests that humans can be implicitly aware of differences in visuomotor maps and adapt accordingly to visual errors. These findings provide suggestions for how to notice and adapt our movements to the environment and our own dynamically changing conditions, to perform accurate movements consistently.

## Graphical abstract

**Figure:**
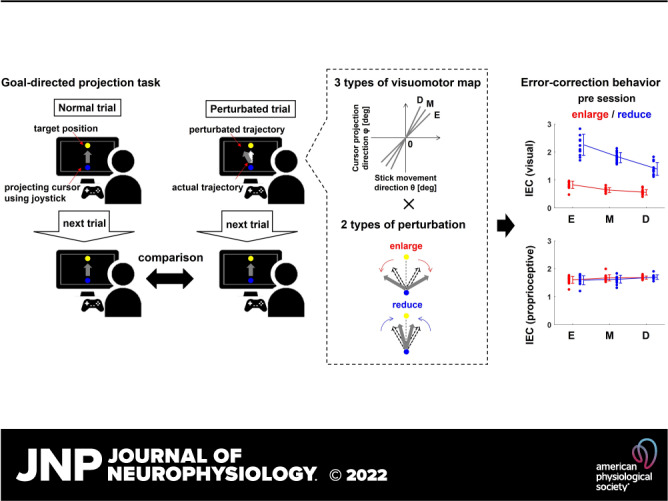

## INTRODUCTION

Humans can transport objects to desired positions out of their reach with some accuracy, by hitting them with tools or throwing them. However, the outcome (the arrival position of the object) of the movement is not always the same. For example, in a sport movement, such as a golf swing, the motor outcome depends on internal and external factors, such as fluctuations in internal neural activation, the equipment used (types of clubs), or the environment (ground and wind); consequently, the outcome inevitably varies, even if the same movement is performed. Therefore, to improve the accuracy of an undesirable outcome, humans correct their movements in the following trial as necessary (1–3), while estimating the relationship between the movement and the outcome based on visual and proprioceptive information during the movement (4–7).

**Figure 1.**
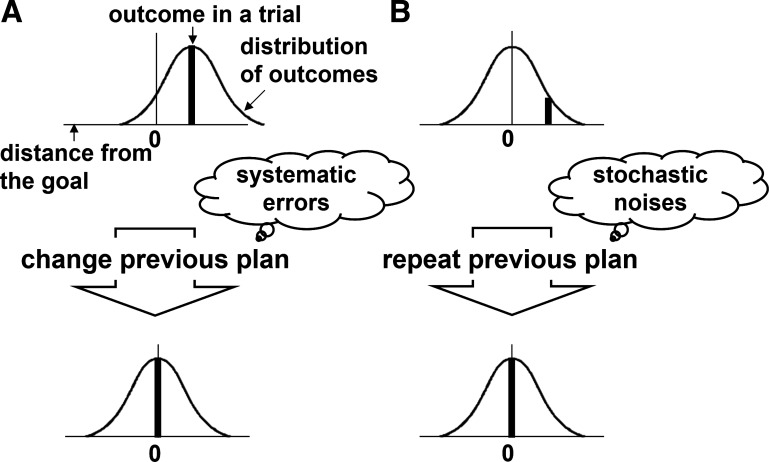
Concept of error correction. If a trial fails, it is necessary to consider whether errors (the causes of the failure) occurred in the planning or execution phase, to correct the errors appropriately in the next planning. *A*: when motor commands are generated in the brain, systematic errors can occur. In this case, the aiming point tends to be biased systematically. *B*: during the relaying of motor commands by neurons and their translation into mechanical force, errors also occur as stochastic noise between planning and execution, because of the added neurophysiological noise. In this case, the results form a probability density (outcome variability), whose average is the aiming point when an ideal motor command (before motor noise is added) is given. We have to change plans in the next trial if we think that the failure is due to systematic errors in the planning phase, or repeat the same plan without correction if we think that the failure occurred as stochastic noise during the execution phase, assuming that the motor outcomes are affected only by ourselves, rather than external factors.

From the perspective of motor control, human movement can be largely divided into two phases: motor planning (8, 9) and motor execution (10–12). Consequently, when the brain generates neural signals and sends them to peripheral muscles, two forms of errors can occur (13). Here, we employed a motor task for a single target used in most previous studies on trial-by-trial error correction. During the task, if there is an error in the planned movement itself, systematic errors can occur. In this case, the results tend to be systematically biased and their center does not match the target position (Fig. 1*A*). in contrast, when relaying signals via neurons and translating these into mechanical force, errors also occur as stochastic noise between planning and execution, because of the added neurophysiological noise. In this case, the results form a probability density (referred to as spread of results) whose average is a result of an ideal motor command (before adding motor noise), i.e., the target position (Fig. 1*B*). Therefore, when facing trial failure, it is necessary to consider what errors (causes of failure) occurred, to appropriately correct these errors the next time (14). In other words, if we attribute the failure to systematic errors, the plan needs to change in the next trial; whereas if we attribute the failure to stochastic noise, the same plan can be repeated without correction, assuming that the errors are not based on planning.

This composition can also be applied to error correction during throwing or hitting under different external factors, as described earlier. For example, an error caused by tools used continuously can be considered as a systematic error and that caused by a temporary wind can be considered as stochastic noise. Therefore, we have to change plans in the next trial if we attribute the failure to the former, and repeat the same plan without correction if we attribute the failure to the latter. In this case, the visuomotor map plays an important role. The visuomotor map refers to the input-output properties of movement (the relationship between movement and outcome), and constructing its model based on vision and proprioceptive has been shown to support motor adaptation (15, 16). If a person is skilled and correctly estimates the visuomotor map, one may ignore temporary visual errors deviating from the conventional relationship between movements and outcomes.

However, this trial-by-trial error-correction has been reported to be mainly based on factors of visual information. One of these is the visual error size (i.e., error between the motor outcome and the target). In the field of motor learning, the processes of motor adaptation with corrections based on visual error are investigated using reaching tasks and are formalized by various models (17, 18). The amount of error corrected from one trial to the next is proportional to the size of the visual error in a small range (19–22), and in a larger range, the sensitivity of error-corrections decreases or saturates (20, 23–25). The interpretation of these results, showing that error-correction behavior changes depend on visual error size (visual error based), was based on the assumption that the environment is usually stable and that only small errors are treated as the source of information that should be used to change planning (26, 27). Here, if “the environment is usually stable,” which is the reason that only small errors are treated, is interpreted as “the large errors rarely occur,” visual errors which are considered not to occur may have been ignored regardless of their size (which means the error correction may have not been visual error-based but visuomotor map-based).

In contrast, a high uncertainty of visual information, such as the uncertain appearance of the position or blurring, reduces the response to errors (27–29). This suggests that, depending on the reliability of the visual information, the degree of its use for error correction may differ. The reliability of visual information also depends on its relationship with other sensory information. In the process of error-correction in many movements, the source of error information available is not only visual error, but also proprioceptive error (i.e., error between the actual proprioceptive feedback and desired proprioceptive feedback hitting the target). A pervious study of how to integrate simultaneously received visual and proprioceptive information showed that the mismatch between the size of visual and proprioceptive errors reduced sensitivity to visual errors (22), and that the size of proprioceptive errors changed the corrective properties to visual error (20). These findings imply the involvement of visuomotor maps, combining multiple sensory information. However, in previous studies investigating the contribution of visual and proprioceptive information by eliminating or disturbing one source of information, vision was still found dominant in motor adaptation studies using reaching tasks (30). Therefore, even in the discrete task of transferring an object to the target, it is also possible that vision remains constantly dominant and that temporary errors deviating from the conventional relationship between movements and outcomes could not be ignored.

Therefore, this study aimed to reveal whether the trial-by-trial error correction in the discrete task of projecting an object to a target is visuomotor map based, not visual error based. We focused on whether *1*) error-correction behavior changed depending on the visuomotor map, and *2*) temporary visual errors deviating from the visuomotor map are ignored (i.e., the visuomotor map is maintained) regardless of the visual error size. To examine *1*), three visuomotor map conditions where the relationship between the actual stick movement (proprioceptive) and cursor projection (visual) differed were employed. To examine *2*), in each condition, the visual information was occasionally perturbed in two ways to create a situation where the visual error was larger or smaller than usual. Here, participants were able to perceive only proprioceptive errors as usual. If the error-correction were constantly visual error-based, participants’ stick movements in the next trial would differ according to perturbation. However, if a robust visuomotor map is constructed (or if the error correction were based on proprioceptive errors), visual perturbation would be ignored, and the actual stick movements would not differ. It has been reported that the degree of correction in response to visual errors changes according to changes in the visuomotor map (31). However, as the visuomotor map was distorted by having only a certain range of movement repeated under different feedback, we used the method which provided a systematic change in outcome variability to investigate the influence of the visuomotor map.

## MATERIALS AND METHODS

### Experiment

**Figure 2.**
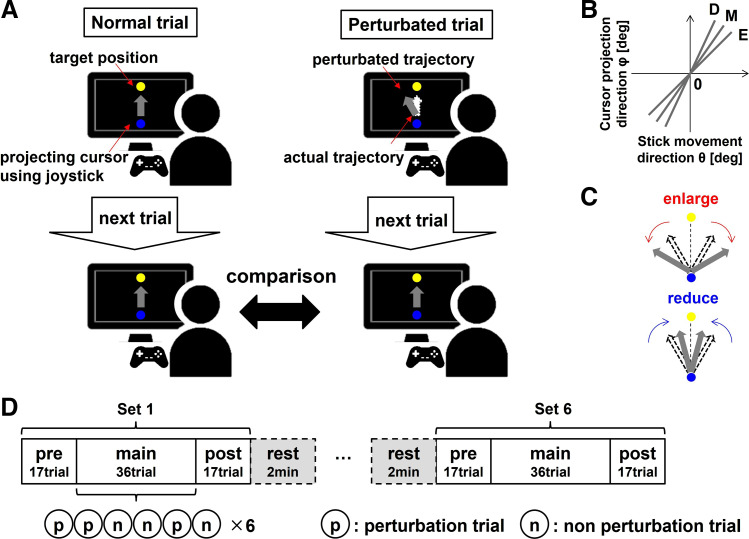
Set up of the experiment. *A*: participants performed the projection task with their right thumb tilting the stick of a game controller without seeing it. They projected the blue circle cursor (5-mm diameter) to the yellow circle target (10-mm diameter) placed 15 cm above the start position, on a screen that was placed in front of them. Perturbations were occasionally used in the middle of the set (main session), in which visual rotations were applied. This setting was established to investigate whether the plan was changed or if the same plan was repeated. *B*: the visuomotor map was defined as the relationship between the actual direction of the stick movement and the direction of the cursor projection. This made it implicitly different for each set as the three levels of the slope of linear relationship (E, M, and D). This setting was established to create a visuomotor map with implicitly different outcome variability. *C*: perturbations, in which visual rotations were applied in the same or opposite direction as the stick tilting, were used. This procedure implicitly made the movement direction of the cursor more distant (i.e., enlarged) or closer (i.e., reduced) even when the participants aimed at the target. This setting was established to compare participants’ correction behaviors in a situation where the visual error was larger or where the proprioceptive error was larger. *D*: seventy trials per set consisted of three sessions: pre session (17 trials), main session (36 trials), and post session (17 trials). In the pre and post sessions, the relationship between the actual direction of stick movement and the cursor projection direction had a linear relationship, and its slope was constant throughout the session. In the main session, there were 6 series of 6 trials with enlarge/reduce perturbation and no perturbation. This setting was established to investigate how the behaviors change in the trial immediately following the trial with one (p1), two (p2), and no (p0) perturbation.

The study was approved by the ethics committee of the University of Tokyo, and all participants provided informed consent. Seventeen right-handed participants (8 males, 9 females; aged 21–45 yr) participated in the experiment. They performed the projection task by using their right thumb to tilt the stick of a game controller (Xbox, Windows) without seeing it. In the experiment, they projected a blue circle cursor (5-mm diameter) to a yellow circle target (10-mm diameter) placed 15 cm above the start position on a screen (size: 286 × 179 mm; resolution: 1,280 × 720 pixels) that was placed in front of them (Fig. 2*A*). When they tilted the stick to 95% of its maximum, the cursor was projected at a speed of 250 mm/s in the direction corresponding to the tilt angle. They could see the trajectory of the cursor and if the cursor hit or missed the target. If the cursor hit the target (the distance between the center of the cursor and target < radius of cursor + radius of target), the color of the target changed to red, and the participant received 100 points. They were instructed to move the stick as quickly and accurately as possible. A warning message “Too slow!” was displayed at the center of the screen if the movement velocity of the stick was slower than 250 mm/s, and participants then did not obtain points. The task was created using MATLAB (MathWorks), and the angle of the stick and the position of the cursor were measured as behavior and feedback at 60 Hz. The data of two participants for which measurement problems occurred were excluded.

The experiment consisted of one practice set and six main sets, and each set consisted of 70 trials. We defined the visuomotor map as the relationship between the actual direction of stick movement (θ) and the cursor projection direction (φ), and changed it in terms of the three slope levels of linear relationship (E:1, M:1.25, D:1.5; Fig. 2*B*) for each set without participants’ knowing. Therefore, the visuomotor map can be described by equation as follows: E:φ = θ, M:φ = 1.25(θ − 90) + 90, D:φ = 1.5(θ − 90) + 90. This setting was established to create a visuomotor map with different motor variabilities. Moreover, we applied a visual rotation to the cursor to enlarge or reduce the error on some trials in the middle of the set (main session; details in the following paragraph) (Fig. 2*C*). This procedure implicitly made the movement direction of the cursor more distant (i.e., enlarged) or closer (i.e., reduced), even when the participants aimed at the target (31, 32). For example, in the enlarge condition, when tilting the stick to the right to the target, rightward visual rotation around the starting position was applied to the cursor and the visual error enlarged. The amount of visual rotation was +(θ − 90) in the enlarge condition, and −(θ − 90)/2 in the reduce condition. Therefore, an error (e.g., 30°) becomes double (60°) in the enlarge condition and becomes half (15°) in the reduce condition. Altogether, it seems the cursor projection direction on any trial is related to the stick movement via: φ = g × [θ + (θ − 90)] on enlarge trials, and φ = g × [θ − (θ − 90)/2] on reduce trials, where g = 1, 1.25, or 1.5 according to the E, M, and D gains. This setting was established to investigate whether the plan was changed or whether the same plan was repeated for the two types of error between trials. Therefore, there were six conditions: three levels of slope of the linear relationship between the actual direction of stick movement (visuomotor map) and the cursor projection direction × two types of perturbation conditions; these conditions were assigned to each set. The order of the sets was randomized among the participants, and participants were not aware of the presence of different visuomotor maps or visual rotations.

Seventy trials per set consisted of three sessions: pre session (17 trials), main session (36 trials), and post session (17 trials). In the pre and post sessions, the relationship between the actual direction of stick movement and the cursor projection direction had a linear relationship, and its slope was constant throughout the session. In the main session, there were six series of six trials with enlarge/reduce perturbation and no perturbation, as shown in Fig. 2*D*. This setting was established to investigate how the behaviors change in the trial immediately following the trial with one (p1), two (p2), and no (p0) perturbation. The rotation applied always enlarge or always reduce in the same set. If visual information were constantly dominant, participants’ actual stick movements would differ in the next perturbation trial and no perturbation trial. On the other hand, if a robust visuomotor map is constructed or if the mismatch between visual and proprioceptive information reduces sensitivity to visual errors, visual perturbation would be ignored to some extent, and the actual stick movements would not differ. Furthermore, to investigate how participants estimated the motor outcomes and their variability, the trajectory of the cursor was made invisible for one out of every seven trials, and participants were asked to answer where the cursor reached. As it is difficult for participants to directly report the cursor projection direction, they were asked to choose the position closest to their own cursor’s arrival position out of a discrete set of cursor positions from 1 to 7. The numbers the participants provided were not hand locations or stick movement directions but the cursor locations on the monitor. Position 4 means an angle to hit the target (90°) and other positions were 5° apart from each other around it, i.e., position 1–7 means 75°–105°.

### Data Analysis

To compare the participants’ behavior for each set, we calculated the standard deviation (SD) of the actual direction of stick movement (θ) and absolute error between θ and an angle to hit the target (90°) for each condition. In addition, to evaluate the error-correction behavior, we examined the amount of correction of the actual direction of stick movement (θ*_n_* − θ*_n_*_−1_) against the visual error of one trial before (φ_n−1_ − 90) and against the proprioceptive errors (θ*_n_*_−1_ − 90). The visual error means the difference between the cursor projection direction (φ) and the angle to hit the target (90°), and the proprioceptive error is the difference between the actual direction of stick movement (θ) and an angle to hit the target (90°). First, linearity in the relation between the amount of correction of the actual direction of stick movement (θ*_n_* − θ*_n_*_−1_) and each error (φ*_n_*_−1_ − 90 or θ*_n_*_−1_ − 90) was assessed, and then by performing regression analysis on the plots of them, we evaluated whether error-correction behavior for visual errors or proprioceptive errors changed across conditions (three visuomotor maps and two perturbation types). The absolute value of the regression line slope was used as an index of error correction (IEC). Therefore, it is conceptually based on the following equation: θ*_n_* − θ*_n_*_−1_ = IEC × (φ*_n_*_−1_ − 90) or (θ*_n_*_−1_ − 90) + const. The larger the index, the more the participant corrects in response to an error.

The IECs were compared between three visuomotor maps and two types of perturbation. If error correction was aimed to correct perturbed visual errors, IECs to visual errors would be constant across conditions, and IECs to proprioceptive errors would change in the direction of the visual perturbation (larger in the enlarge condition and smaller in the reduce condition) and would also differ according to three visuomotor maps. However, if a robust visuomotor map is constructed and visual perturbation is ignored to some extent, IECs to proprioceptive errors would be constant across conditions, and IECs to visual errors would change to cancel out the visual perturbation (be smaller in the enlarge condition and larger in the reduce condition) and would also differ according three visuomotor maps. Moreover, to investigate how the error-correction behavior changed in the trial immediately following the trial with one (p1), two (p2), and no (p0) perturbations, we compared the IEC for each trial in the main session.

Furthermore, to investigate how participants estimated the motor outcomes and their variability, we compared the SD of the answer of estimation (converted to stick movement direction) and that of the actual direction of stick movement (θ).

SD of θ and absolute error between an angle to hit the target (90°) and θ were compared between three visuomotor maps (E, M, and D) and two types of perturbation (enlarge and reduce) using a two-way repeated-measures analysis of variance (ANOVA). This statistical analysis was also performed for the comparison of error-correction behavior (IECs). To investigate how participants estimated the motor outcomes and their variability, we compared the SD of the answer of estimation and that when participants choose the closest position to the actual movement direction. To determine if the former value subtracted by the latter value was different than 0, a one-sample *t* test was used. Statistical significance was delineated at *P* < 0.05 throughout the study.

## RESULTS

### Stick Movement Direction for Each Set

To reveal the integration of visual and proprioceptive information in goal-directed projection tasks in which visuomotor maps are thought to be important, we compared participants’ error-correction behaviors in the situation where the relationship in size between two errors is created by visual perturbation, under three conditions in which the visuomotor map differed implicitly.

**Figure 3.**
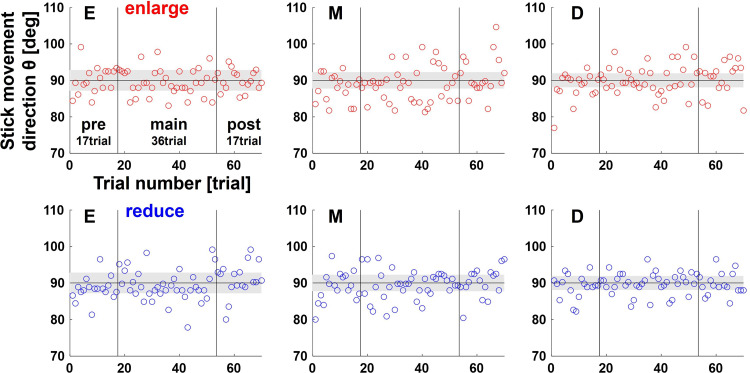
The time series of the actual direction of stick movement of a typical participant. The plots show the time series of the actual direction of stick movement for each set for a typical participant. Colored areas represent the range of values at which the cursor must be to hit the target (achieve success).

**Figure 4.**
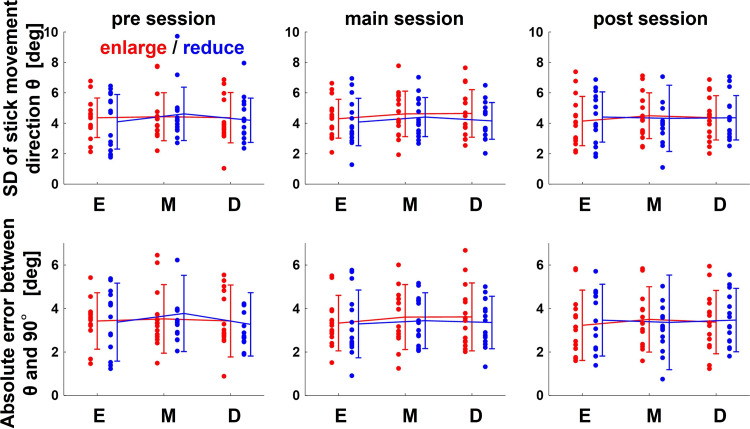
The average standard deviation (SD) and the absolute error of the actual direction of stick movement for all participants for each set. The line graphs show the averages of all participants, and the data points show each individual participant’s value. Error bars indicate standard deviation. Two-way repeated-measures analysis of variance (ANOVA) for the SD and absolute error in pre sessions, main sessions, and post sessions did not show any main effect of visuomotor map or of perturbation type.

The average scores (success rates) of all participants for each set were E: 39.8 ± 12.1, M: 35.0 ± 15.2, D: 26.7 ± 14.9 in the enlarge condition, and E: 53.5 ± 16.2, M: 41.1 ± 11.5, D: 41.1 ± 12.8 in the reduce condition. It decreased as the slope of the linear relationship between the actual direction of stick movement and the cursor projection direction increased (i.e., as the variability of the latter relative to that of the former increased), and was lower in the enlarge condition than in the reduce condition. These were reasonable results given the characteristics of the visuomotor map and perturbation. However, comparing the actual direction of stick movement for each set, there were no significant differences in the participants’ behavior in each condition. Figure 3 shows the time series of the actual direction of stick movement of a typical participant, and Fig. 4 shows the average SD and the absolute error of the actual direction of stick movement for each set. Comparing the SD and the absolute error of the actual direction of stick movement between conditions for each session, neither main effect nor interaction was found. Two-way repeated-measures analysis of variance (ANOVA) for the SD in pre sessions [visuomotor map: *F*(2,84) = 0.29, *P* = 0.75; perturbation: *F*(1,84) = 0.06, *P* = 0.81; visuomotor map × perturbation interaction *F*(2,84) = 0.17, *P* = 0.85], main sessions [visuomotor map: *F*(2,84) = 41, *P* = 0.67; perturbation: *F*(1,84) = 1.06, *P* = 0.31; visuomotor map × perturbation interaction *F*(2,84) = 0.10, *P* = 0.91], and post sessions [visuomotor map: *F*(2,84) = 0.48, *P* = 0.95; perturbation: *F*(1,84) = 0.01, *P* = 0.92; visuomotor map × perturbation interaction *F*(2,84) = 0.13, *P* = 0.88] and the absolute error in pre sessions [visuomotor map: *F*(2,84) = 0.43, *P* = 0.65; perturbation: *F*(1,84) = 0.00, *P* = 0.96; visuomotor map × perturbation interaction *F*(2,84) = 0.19, *P* = 0.83], main sessions [visuomotor map: *F*(2,84) = 0.25, *P* = 0.78; perturbation: *F*(1,84) = 0.35, *P* = 0.56; visuomotor map × perturbation interaction *F*(2,84) = 0.06, *P* = 0.94] and post sessions [visuomotor map: *F*(2,84) = 0.04, *P* = 0.97; perturbation: *F*(1,84) = 0.05, *P* = 0.82; visuomotor map × perturbation interaction *F*(2,84) = 0.15, *P* = 0.86] did not show any main effect of visuomotor map or of perturbation type.

### Error-Correction Behavior for Each Set

**Figure 5.**
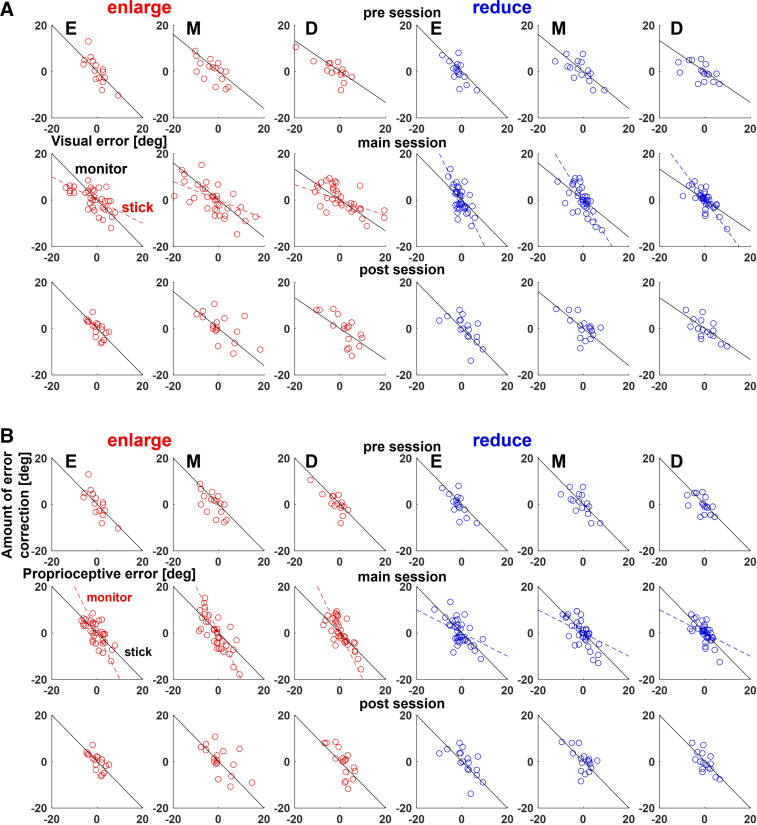
The amount of correction of stick movement against error in one trial before. *A*: the scatter plots show the amount of correction of stick movement against visual error in one trial before for each set of a typical participant. The two identical lines (straight and broken) in a main session show the case of error correction for the actual direction of stick movement and the cursor projection direction, respectively. *B*: the scatter plots show the amount of correction of stick movement against proprioceptive error in one trial before for each set of a typical participant. The two identical lines (straight and broken) in a main session show the case of error correction for the cursor projection direction and the actual direction of stick movement, respectively.

To evaluate the error-correction behavior, we examined the actual direction of stick movement against the visual error of one trial before. Figure 5*A* shows the scatter plots of the amount of correction of the actual direction of stick movement in the following trial for the visual error of one trial before for each set of a typical participant. In the figure, the two identical lines (straight and broken) in the main session show the case of error correction for the cursor projection direction (visual) and the actual direction of stick movement (proprioceptive), respectively. As linearity in the relation between them was assessed (*P* < 0.01), regression analysis was performed on the plots, and absolute value of the regression line slope was used as an IEC to evaluate correction to visuomotor map and visual perturbations. The larger the index, the more the participant corrects in response to an error.

**Figure 6.**
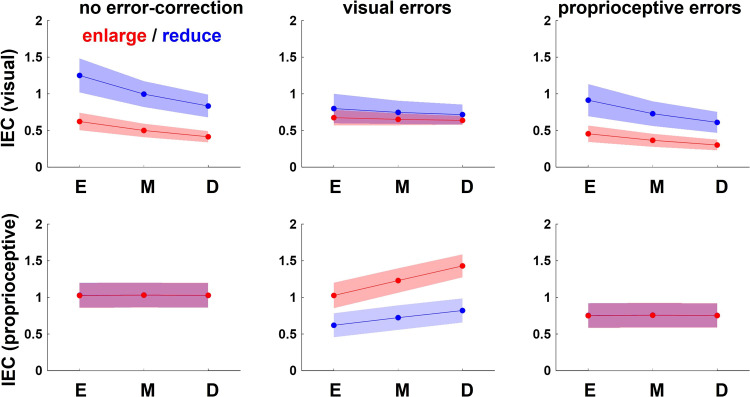
Simulation of index of error correction (IEC). A model, where the error-correction behavior is updated from trials *n* to *n* + 1 according to an error could be used to simulate when participants corrected the actual direction of stick movement (θ) to a visual error, a proprioceptive error, or some combination of the two, and to illustrate how IEC should change between cases. We compared the observed data to the simulations of IEC using a model: θ*_n_*_+1_ = aθ*_n_* – nothing or b(φ*_n_* − 90) or b(θ*_n_* − 90) + N(0,10), where a represents a retention factor and b represents an error sensitivity and N(0,10) represents noise from a normal distribution with a mean of 0°and a standard deviation of 10°. The value of a was set to 1 and that of b was set to 0.5 throughout all models. If no error correction was performed or a robust visuomotor map was constructed and visual perturbations were ignored to some extent, IECs to visual errors would change to the opposite to the visual perturbation (be smaller in the enlarge condition and larger in the reduce condition) and also differ according three visuomotor maps (*top right* and *left*), and IECs to proprioceptive errors would be constant across the conditions (*bottom right* and *left*). If error correction was for perturbed visual errors, IECs to visual errors would be constant across conditions (*top center*) and IECs to proprioceptive errors would change in the direction of the visual perturbation (be larger in the enlarge condition and smaller in the reduce condition) and also differ according three visuomotor maps (*bottom center*).

Before presenting the result of IECs, it was considered how IEC in each condition will change in three extremes: *1*) participants do not correct errors and just repeat the same movements, *2*) participants correct only visual errors, and *3*) participants correct only proprioceptive errors. This was performed using a model (e.g., x*_n_*_+ 1_ = ax*_n_* + be*_n_*, where the behavior x is updated from trials *n* to *n* + 1 according to an error e, and a and b are a retention factor and error sensitivity, respectively), which describes how behavior changes in response to error. Such a model could be used to simulate when participants did not correct errors and when participants corrected the actual direction of stick movement (θ*_n_*_+ 1_) to a visual error (φ*_n_* − 90) and a proprioceptive error (θ*_n_* − 90), and to illustrate how IEC should change across cases. We simulated IEC using three models: *1*) θ*_n_*_+ 1_ = N(90, σ), *2*) θ*_n_*_+ 1_ = aθ*_n_* – b(φ*_n_* − 90) + N(0, σ), and *3*) θ*_n_*_+ 1_ = aθ*_n_* – b(θ*_n_* − 90) + N(0, σ), where a represents a retention factor, b represents an error sensitivity, and N(0, σ) represents noise from a normal distribution with a mean of 0°and an SD of σ°. The value of a was set to 1 and that of b was set to 0.5 throughout all models. Figure 6 shows how IEC will change in each condition in each model. Therefore, if no error correction was performed or a robust visuomotor map was constructed and visual perturbations were ignored to some extent, IECs to visual errors would change to the opposite to the visual perturbation (be smaller in the enlarge condition and larger in the reduce condition) and also differ according three visuomotor maps (*top right* and *left* in Fig. 6), and IECs to proprioceptive errors would be constant across the conditions (*bottom right* and *left* in Fig. 6). If error correction was for perturbed visual errors, IECs to visual errors would be constant across conditions (*top center* in Fig. 6) and IECs to proprioceptive errors would change in the direction of the visual perturbation (be larger in the enlarge condition and smaller in the reduce condition) and also differ according three visuomotor maps (*bottom center* in Fig. 6). Comparing the observed data to these simulated IECs can aid with interpretation of the data.

**Figure 7.**
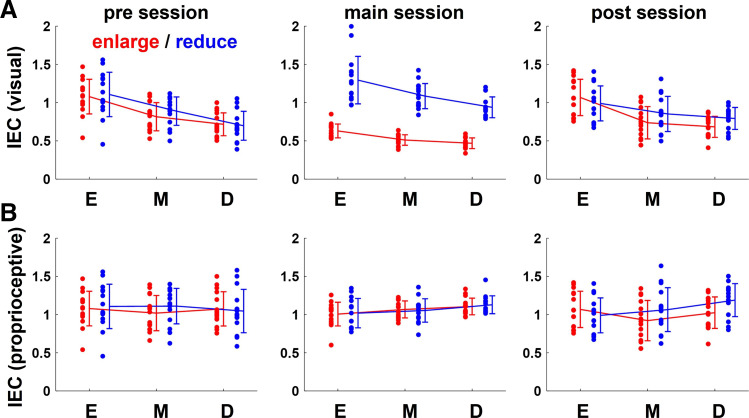
The index of error correction (IEC) using regression analysis. *A*: regression analysis was performed on plots for visual error, and the absolute value of the regression line slope was represented as the index of error correction (IEC). The graphs show the average of this index of all participants for each set. The line graphs show the average of all participants and the data points show each individual participant’s value. Error bars indicate SDs. In the main sessions, it changed in the direction of under-correction in the enlarge condition and in the direction of over-correction in the reduce condition. Two-way repeated-measures ANOVA for the IECs in main sessions showed a main effect of visuomotor map and perturbation type [visuomotor map: *F*(2,84) = 19.23, *P* < 0.01; perturbation: *F*(1,84) = 274.23, *P* < 0.01; visuomotor map × perturbation interaction *F*(2,84) = 2.68, *P* = 0.07] while that in pre sessions [visuomotor map: *F*(2,84) = 26.04, *P* < 0.01; perturbation: *F*(1,84) = 0.38, *P* = 0.53; visuomotor map × perturbation interaction *F*(2,84) = 0.37, *P* = 0.69] and post sessions [visuomotor map: *F*(2,84) = 17.42, *P* < 0.01; perturbation: *F*(1,84) = 1.32, *P* = 0.25; visuomotor map × perturbation interaction *F*(2,84) = 2.22, *P* = 0.12] did not show that of perturbation type. *B*: regression analysis was performed on plots for proprioceptive error, and the absolute value of the regression line slope, was represented as the index of error correction (IEC). The graphs show the average of this index of all participants for each set. The line graphs show the average of all participants and the data points show each individual participant’s value. Error bars indicate SDs. Participants consistently overcorrected across the sets in pre sessions and post sessions. In the main sessions, it changed in the direction of under-correction in the enlarge condition and in the direction of over-correction in the reduce condition. Two-way repeated-measures ANOVA for the IECs for each session did not show any main effect of visuomotor map or of perturbation type.

Figure 7*A* shows the average of IECs to visual errors for all participants for each set. It indicates that error-correction behavior changed in the direction of under correction in the enlarge condition and in the direction of over correction in the reduce condition. Two-way repeated-measures ANOVA showed a main effect of perturbation type only in main sessions [visuomotor map: *F*(2,84) = 19.23, *P* < 0.01; perturbation: *F*(1,84) = 274.23, *P* < 0.01; visuomotor map × perturbation interaction *F*(2,84) = 2.68, *P* = 0.07]. Therefore, the error-correction behavior to visual errors changed opposite to that to visual perturbation, which seemed to be because the participants ignored visual perturbation.

The same analysis was performed for proprioceptive errors. Figure 5*B* shows the scatter plots of the amount of correction of the actual direction of stick movement in the next trial for the proprioceptive error of one trial before for each set of a typical participant. The two identical lines (straight and broken) in the main session show the case of error correction for the actual direction of stick movement (proprioceptive) and the cursor projection direction (visual), respectively. Two-way repeated-measures ANOVA of the IECs to proprioceptive errors in all sessions showed neither main effect nor interaction (Fig. 7*B*). This indicates that participants did not change their error correction for proprioceptive error even under visual perturbations.

**Figure 8.**
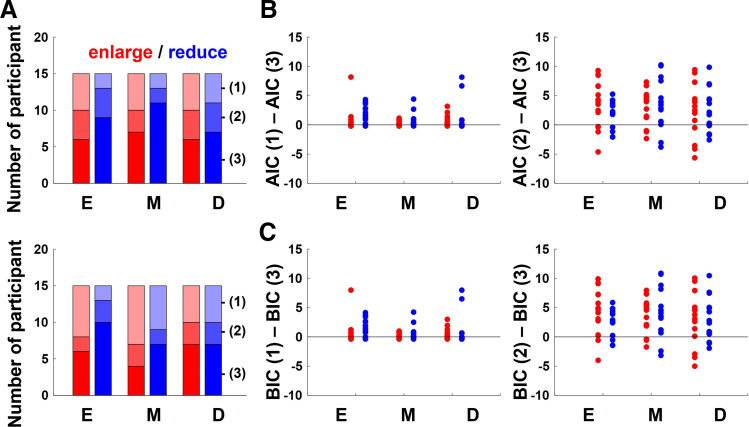
The AIC and BIC for each model. *A*: since the index of error corrections (IECs) of the observed data were similar to those when there were either no error corrections or when the error-correction patterns were based on proprioceptive error, we used a formal comparison of models with AIC and BIC for validation. The model which best describes the data (i.e., minimizes AIC or BIC) for most participants was regarded as the one most likely to describe the data. The graphs show the number of participants whose data was best described by each model. *B*: the relative values for AIC and BIC for *models 1* and *2* subtracted by that for the best-performing *model 3* for each participant were calculated. Positive values indicate that AIC or BIC for the alternate model was greater, and that the alternate model is less likely to explain the data. The line graphs show the average the relative AIC of all participants, and the data points show each individual participant’s value. Error bars indicate standard deviation. *C*: a similar analysis was performed for BIC. The line graphs show the average the relative BIC of all participants, and the data points show each individual participant’s value. Error bars indicate standard deviation.

To summarize, given that the IECs of the observed data were similar to those of right or left in Fig. 6, it was found that there were either no error corrections or error-correction patterns were based on proprioceptive error. Therefore, we used a formal comparison of models with Akaike information criterion (AIC) and Bayesian information criterion (BIC) to determine the superiority of the models. The proprioceptive model: *3*) θ*_n_*_+ 1_ = aθ*_n_* – b(θ*_n_* − 90) + N(0, σ) can be transformed to θ*_n_*_+ 1_ = (a − b)θ*_n_* + N(90b, σ) and it is similar to the no error-correction model *1*) when a = 1 and b = 1. This means the problem whether the no error correction and the proprioceptive model resembled the data more is at which value of b the model resembled the data most. Therefore, first, the three models were fitted to each individual participant’s data. We started with an individual participant’s model with the noise term’s SD (σ) being unfixed: *1*) θ*_n_*_+ 1_ = N(90, σ), *2*) θ*_n_*_+ 1_ = aθ*_n_* – b(φ*_n_* − 90) + N(0, σ), *3*) θ*_n_*_+ 1_ = aθ*_n_* – b(θ*_n_* − 90) + N(0, σ), where a was set to 1 and was treated as a known parameter in the proprioceptive and visual error models, and the only unknowns were σ and b. The noise terms were ignored and the b value that minimizes the squared error between the simulated and the observed direction of stick movement, i.e., SD of the residual, in main session were found. Once b was identified, σ were calculated as the SD of the residual. With σ and b for each participant, each model’s log-likelihood was used to calculate AIC and BIC. The no error correction model had only one parameter (σ only) to penalize in each expression and the proprioceptive and visual error models had two parameters to penalize (σ and b). The model which best describes the data (i.e., minimizes AIC or BIC) for most participants was regarded as the one most likely to describe the data. As a result, the proprioceptive *model 3* best described the participants’ data in all conditions (11 participants’ data in M in the reduce condition, 9 participants’ data in E in the reduce condition, 7 participants’ data in M in the enlarge condition and D in the reduce condition, and 6 participants’ data in E and M in the enlarge condition) in AIC. On the other hand, the no error-correction *model 1* best described the participants’ data in E and M in enlarge conditions (8 participants’ data in M and 7 participants’ data in E in the enlarge condition), although the proprioceptive *model 3* best described the participants’ data in other conditions (10 participants’ data in E in the reduce condition and 7 participants’ data in D in the enlarge condition and in M and D in the reduce condition) in BIC. Therefore, overall, it was concluded that the error correction patterns were based on a proprioceptive error. Figure 8*A* shows the number of participants whose data were best described by each model, and Fig. 8, *B* and *C* shows the relative values for AIC and BIC for *models 1* and *2* while subtracting *model 3* in each participant, respectively. The positive values here mean that AIC or BIC for the alternate model was greater, and thus the alternate model is less likely to explain the data.

### Error-Correction Behavior for Each Trial

**Figure 9.**
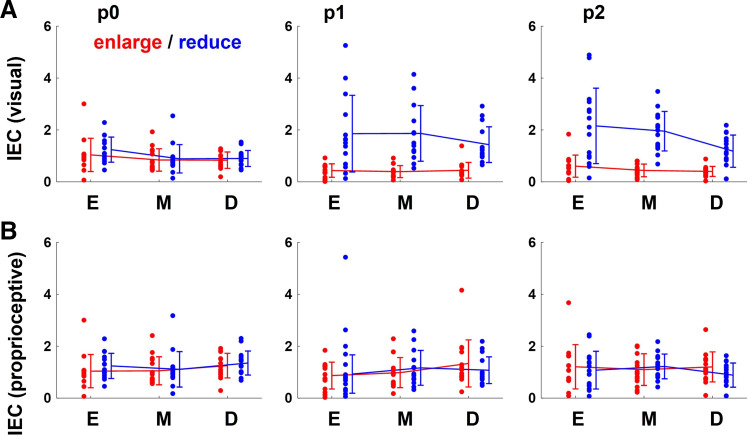
The index of error correction (IEC) for each trial. *A*: to investigate how the behaviors change in the trial immediately following the trial with one (p1), two (p2), and no (p0) perturbation, the variability of the actual direction of stick movement for each was compared. The line graphs show the average the index of error correction (IEC) of all participants, and the data points show each individual participant’s value. Error bars indicate standard deviation. Participants consistently overcorrected across the sets in the pre and post sessions. In the main sessions, it changed in the direction of under-correction in the enlarged condition and in the direction of over-correction in the reduce condition. A two-way repeated-measures ANOVA for the results p2 [visuomotor map: *F*(2,84) = 4.85, *P* < 0.05; perturbation: *F*(1,84) = 66.02, *P* < 0.01; visuomotor map × perturbation interaction *F*(2,84) = 2.53, *P* = 0.09] and p1 [visuomotor map: *F*(2,84) = 1.25, *P* = 0.29; perturbation: *F*(1,84) = 33.6, *P* < 0.01; visuomotor map × perturbation interaction *F*(2,84) = 1.30, *P* = 0.28] showed a main effect of visuomotor map and perturbation type, whereas that in p0 [visuomotor map: *F*(2,84) = 3.38, *P* < 0.05; perturbation: *F*(1,84) = 1.11, *P* = 0.29; visuomotor map × perturbation interaction *F*(2,84) = 0.25, *P* = 0.78] did not show that of perturbation type. *B*: similar analysis was performed for proprioceptive errors. The line graphs show the average the index of error correction (IEC) of all participants, and the data points show each individual participant’s value. Error bars indicate standard deviation. Participants consistently overcorrected across the sets in all sessions. Even in the main sessions, it did not change. A two-way repeated-measures ANOVA for each session did not show any main effect of visuomotor map or of perturbation type.

To investigate how the error-correction behavior changed in the trial immediately following the trial with one (p1), two (p2), and no (p0) perturbation, the IEC for each trial in the main session was compared. Figure 9*A* and Fig. 8*B* show the average values to visual errors and proprioceptive errors of all participants for each set. Comparing the IEC between conditions for each session, we found a main effect of perturbation type in p2 [visuomotor map: *F*(2,84) = 4.85, *P* < 0.05; perturbation: *F*(1,84) = 66.02, *P* < 0.01; visuomotor map × perturbation interaction *F*(2,84) = 2.53, *P* = 0.09] and p1 [visuomotor map: *F*(2,84) = 1.25, *P* = 0.29; perturbation: *F*(1,84) = 33.6, *P* < 0.01; visuomotor map × perturbation interaction *F*(2,84) = 1.30, *P* = 0.28] but not in p0 [visuomotor map: *F*(2,84) = 3.38, *P* < 0.05; perturbation: *F*(1,84) = 1.11, *P* = 0.29; visuomotor map × perturbation interaction *F*(2,84) = 0.25, *P* = 0.78], in IEC to visual errors. This means that the IEC changed in the direction of under-correction in the enlarge condition and in the direction of over-correction in the reduce condition only in the trial immediately following the trial with perturbation. On the other hand, IEC to proprioceptive errors did not change even in the trial immediately following the trial with perturbation. If data plots are distributed almost isotopically or their number is too small, there will be almost no difference in the variances in any directions. In this case, regression analysis may result in various values for those not appropriate for capturing the characteristics of the data. Therefore, in this study values of IEC too far from 1 (greater than30 or less than −30) were not used in subsequent statistical analyses.

### Estimation of Motor Outcomes

**Figure 10.**
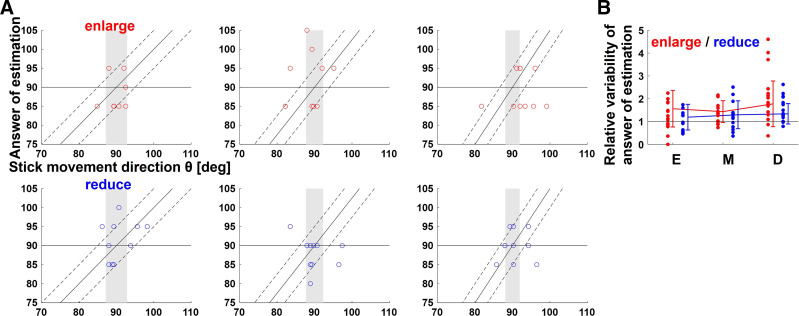
Estimation of motor outcome. *A*: to investigate how participants estimated motor outcomes and their variability, the trajectory of the cursor was made invisible for one out of every seven trials, and participants were asked to answer where the cursor reached. They chose the position closest to their own cursor’s arrival position out of a discrete set of cursor positions from 1 to 7. The scatter plots show the answer of estimation against the actual direction of stick movement for each set of a typical participant. The three identical lines (one straight and two broken) show correct estimation, and its upper and lower limits, respectively. Colored areas represent the range of values at which the cursor must be to hit the target (achieve success). *B*: the line graphs show the average of variability the answer of estimation (converted to stick movement direction) divided by that of the actual direction of stick movement for all participants, while the data points show each individual participant’s value. Error bars indicate standard deviations. The one-sample *t* test, used to determine if the estimated value was greater than the actual value, shows that values of M and D in enlarge conditions were significantly smaller and those of the other conditions were not significantly different [E: M = −0.12, *t*(14) = −1.00, *P* = 0.33; M: M = −0.39, *t*(14) = −3.90, *P* < 0.01; D: M = −0.47, *t*(14) = −3.63, *P* < 0.01; in enlarge condition; E: M = −0.02, *t*(14) = −0.23, *P* = 0.82; M: M = −0.02, *t*(14) = −2.06, *P* = 0.06; D: M = −0.03, *t*(14) = −2.11, *P* = 0.05; in reduce condition].

To investigate how participants estimated the motor outcomes and their variability, the trajectory of the cursor was made invisible for one out of every seven trials and participants were asked to answer where the cursor reached. The SD of the answer of estimation and of when participants chose the closest position to the actual movement direction was compared. To determine if the former value subtracted by the latter value was different than 0, a one-sample *t* test was used. As a result, values of M and D in enlarge conditions were significantly smaller than 0 and those of the other conditions were not significantly different than 0 [E: M = −0.12, *t*(14) = −1.00, *P* = 0.33; M: M = −0.39, *t*(14) = −3.90, *P* < 0.01; D: M = −0.47, *t*(14) = −3.63, *P* < 0.01; in enlarge condition; E: M = −0.02, *t*(14) = −0.23, *P* = 0.82; M: M = −0.02, *t*(14) = −2.06, *P* = 0.06; D: M = −0.03, *t*(14) = −2.11, *P* = 0.05; in reduce condition]. This indicates participants underestimated their outcome variabilities in M and D in enlarge conditions and estimated almost correctly in the other conditions. Figure 10*A* shows the estimation response against the actual direction of stick movement of a typical participant, and Fig. 10*B* shows the average variability of the answer of estimation subtracted by when participants chose the closest position to the actual movement direction.

## DISCUSSION

### Implicit Response to Visuomotor Map and Perturbations

This study investigated the error-correction behaviors to visual errors and focused on whether temporary visual errors deviating from the conventional relationship between movements and outcomes are ignored. We compared participants’ correction behaviors in situations where the visual error was larger or where the proprioceptive error was larger, as a result of visual perturbation, under three conditions where the visuomotor map implicitly differed. The results showed that the participants’ error-correction behavior (IEC) to visual errors changed between perturbation types. However, the change was not in the direction of the visual perturbation, but rather the direction of canceling out the visual perturbation (Fig. 7*A*). Moreover, IEC to proprioceptive errors did not change even in main sessions (Fig. 7*B*). These changes of IECs were like the simulated IECs when error-correction patterns were based on a proprioceptive error (*right* in Fig. 6). This is thought to be the result of the participants corrected errors constantly according to visuomotor map (proprioceptive errors), not according to the visual error size.

Our interpretation of the reasons for this is that the participants maintained the visuomotor map constructed in the pre session in the main session and corrected errors based on the stick movement direction in all conditions. This is supported by the finding that IEC did not change between visuomotor maps in all sessions which suggests participants’ error-correction behavior maintained in a constant relationship to visuomotor map. This suggests that they were implicitly aware of the differences in the visuomotor maps and changed their correction to visual errors accordingly in the pre session. Moreover, since the same trend was maintained in the main session, the results may imply that visual perturbations are implicitly noticed due to the constructed visuomotor maps and that temporary visual errors were ignored. This interpretation was supported by the finding that IEC to visual error changed only in the trial immediately following the trial with perturbation (Fig. 9*A*). It has been previously reported that the sensitivity of error correction is proportional to the size of the visual error in a small range and decreases or saturates in a large range (21, 24). If such correction is made, the sensitivity of visual error correction would decrease only in the enlarge condition of this study, and would not be changed in the reduce condition where the visual error is small. In such case, IEC to visual error in the reduce condition should be about the same in the trial immediately following the trial with or without perturbation, but the actual IEC is larger in the trial immediately following the trial with perturbation. This supports the idea that the results of this study cannot be explained by mere changes in error correction to increases or decreases in visual error size, but rather by using visuomotor maps.

In the experimental protocol of this study, IECs to visual errors would show similar change when no error correction was performed and when a robust visuomotor map was constructed while ignoring visual perturbations (Fig. 6). Although we used a formal comparison of models with AIC and BIC and concluded the proprioceptive model, which best describes the data for most participants, is the best, it is still possible that some participants in certain conditions just repeated the same movements without error correction. The no error-correction *model 1* best described the participants’ data in E and M in the enlarge conditions, although the proprioceptive *model 3* best described the participants’ data in other conditions in BIC. The possibility that some participants exhibit no correction would be consistent with a previous study which suggests that error due to internally generated noise does not contribute to the learning process. To clarify this situation, it is possible to investigate error-correction behavior to proprioceptive perturbation similarly. However, in any case, the present findings still showed that the temporary visual errors deviating from the visuomotor map can be ignored regardless of the size of the visual and proprioceptive errors.

### Implications from Findings for Related Fields

In our daily lives, the environment and our own conditions change dynamically, and to perform the movements correctly, we need to notice changes and adapt our movements accordingly. In this study, we found that participants changed their responses to the visual errors under different visuomotor maps and the possibility of the ability to be implicitly aware of differences in visuomotor maps rather than reacting to visual perturbation. The results support the finding that the nervous system estimates the relevance of each observed error and adapts only to relevant errors (22). Moreover, participants were probably able to perceive the difference in outcome variability between sets at the beginning of each set, due to the visuomotor map. Context-specific adaptation is more likely to occur quickly when errors are due to external interfaces (visuomotor rotation and force fields) than when they are due to one’s own sensorimotor performance (prism and Coriolis) (33). In this study, where visuomotor rotation and discrete projection tasks were used, it could be thought that more attention was paid to the environment and that adaptation occurred rapidly (in only 17 trials). This protocol may be useful for adaptation studies, although whether this rapid adaptation is robust, even if the change in the visuomotor map is in the middle of a set, needs to be investigated in future.

A possible reason for the maintenance of the visuomotor map is that, for the error correction, proprioceptive information was integrated to contribute more than visual information. It has been reported that vision is dominant in motor adaptation, even though a mismatch between visual and proprioceptive information occurs (30). The inconsistency of this with the findings of this study may be because most of the tasks used in motor learning are reaching tasks. For the representation of the body in space, proprioceptive information is reportedly the dominant sensory input, and the distinction between movements made to a target in space, as coded in an extrinsic coordinate system, and that made to the relationship between body parts, as coded in an intrinsic coordinate system for motor planning, may be related to this difference (34). The task used in this study (tilting the invisible joystick to move the cursor to the target position) may involve the latter proprioception-based coordinate system, while the reaching task requiring actual body movement to a visual target may involve the former, visual-based coordinate system. Therefore, it was suggested that, even for the same goal-directed task, characteristics such as the object being controlled or the use of tools may also influence multisensory integration or error-correction behavior. Moreover, although in this study we used the motor task for a single target used in most previous studies on error correction, it is necessary to consider redundancy in the solution. If there are some regions where we can achieve the goal of state space, there is the possibility that planning is changed to avoid noisier regions, for example. How multisensory integration and error-correction behavior change in such a case should be investigated in the future.

Moreover, there have been reports related to the estimation of motor outcome in motor decision making and motor learning studies. It has been shown that there are cognitive biases in the estimation of outcome variability, and that participants sometimes underestimate their outcome variability or estimate its isotropy (35–39). The results of this study, revealed that participants underestimated their outcome variabilities in M and D in enlarge conditions and estimated almost correctly in the other conditions. One previous study reported that participants were able to estimate the outcome variability of others (extrinsic factor) accurately, but underestimated their own outcome variability (intrinsic factor) (37). The results of the present study can be interpreted as indicating that participants did not attribute the change in outcome variability to stochastic noise and can be considered to be supportive of these prior findings. The participants may have attributed visual errors to the visuomotor map (extrinsic factor), which can be estimated relatively correctly to a source of motion variability, and underestimated their stochastic noise (intrinsic factor).

### Future Issues

How humans correct errors has been investigated in the context of adaption or response to new tasks or environments created by various tools, which change the feedback the executors can receive (1, 40). One approach is to use devices that create virtual reality environments, such as the one used in this study, particularly computer displays in which the position of the body or the end effector is indicated by a cursor and cannot be seen directly (41, 42). In these studies, the visual feedback can be manipulated (often by rotation in a polar reference frame). Thus, error correction has been investigated as a trial-by-trial change in movements under perturbated visual feedback and that in which perturbation has been removed (after effect). Here, we present two issues related to the methods used to investigate error correction in this study that should be considered in future.

The first issue was whether the participants were trying to correct the error. Since participants were instructed to perform the movements correctly (to score well), it is implicitly assumed that any changes in behavior are the result of attempts at corrections, to adapt to the environment. However, humans are sometimes misguided and have cognitive biases, and movements and outcomes do not always reflect participants’ intentions. For example, in basketball shooting, players who have made a successful shot are more likely to make it again on the next attempt (hot hand), but shooting results do not necessarily provide the evidence (43). This means that participants’ intentions and outcomes do not always correspond. In addition, participants in this study were not aware of any changes in visuomotor map or perturbations in the first place (there was no verbal report); thus, it is difficult to consider whether they corrected errors “intentionally,” even if they really were intentional. Although humans learn and adapt both explicitly and implicitly (44–49), the contribution of error correction of motor planning to these processes requires further investigation.

The second issue is the index for quantifying error correction. In this study, we mainly used the difference in the behaviors for each condition as the index, but this is only a relative evaluation. Moreover, it is not clear how much different the value needs to be to be regarded as representing error correction. As the significance of the difference depends on various factors related to the task and the context, such as reward, it is not immediately determinable. It will be necessary to examine how to determine error correction that may only be shown through comparison, as this is a major issue that concerns the definition of the concept of error correction. Furthermore, it is highly possible that not only the previous trial but also the trials before that are used to make decisions on correction of motor planning, or at least to construct a visuomotor map. The number of previous trials yielding information that is considered for these processes should be investigated in future.

### Conclusions

By investigating the error-correction behaviors to visual errors under different visuomotor maps, and particularly the integration of visual and proprioceptive information, this study revealed that temporary visual errors deviating from the conventional relationship between movements and outcomes can be ignored. We compared participants’ correction behaviors for two types of perturbation under three conditions, where the relationship between the actual joystick movement direction and cursor projection direction on the monitor differed covertly. Participants changed their responses to the visual errors, which suggested that they were implicitly aware of differences in visuomotor maps and corrected errors accordingly. These findings provide suggestions for how to notice and adapt our movements to the environment and our own dynamically changing conditions, to perform accurate movements consistently.

## GRANTS

This work was in part supported by Japan Science and Technology Agency (JSPS KAKENHI 20H04069).

## DISCLOSURES

No conflicts of interest, financial or otherwise, are declared by the authors.

## AUTHOR CONTRIBUTIONS

A. Kusafuka, R.O., and A. Kimura conceived and designed research; A. Kusafuka performed experiments; A. Kusafuka analyzed data; A. Kusafuka, R.O., and A. Kimura interpreted results of experiments; A. Kusafuka prepared figures; A. Kusafuka drafted manuscript; R.O. and K.K. edited and revised manuscript; A. Kusafuka, R.O., A. Kimura, and K.K. approved final version of manuscript.
